# Public Opinion Propagation Prediction Model Based on Dynamic Time-Weighted Rényi Entropy and Graph Neural Network

**DOI:** 10.3390/e27050516

**Published:** 2025-05-12

**Authors:** Qiujuan Tong, Xiaolong Xu, Jianke Zhang, Huawei Xu

**Affiliations:** 1 School of Science, Xi’an University of Posts and Telecommunications, Xi’an 710121, China; tongqiujuan@xupt.edu.cn (Q.T.); jiankezhang@xupt.edu.cn (J.Z.); 2 School of Communication and Information Engineering, Xi’an University of Posts and Telecommunications, Xi’an 710121, China; xuahuwei@stu.xupt.edu.cn

**Keywords:** dynamic time-weighted Rényi entropy, graph neural networks, spatiotemporal fusion modeling, node embeddings, public opinion prediction

## Abstract

Current methods for public opinion propagation prediction struggle to jointly model temporal dynamics, structural complexity, and dynamic node influence in evolving social networks. To overcome these limitations, this paper proposes a public opinion dissemination prediction model based on the integration of dynamic time-weighted Rényi entropy (DTWRE) and graph neural networks. By incorporating a time-weighted mechanism, the model devises two tiers of Rényi entropy metrics—local node entropy and global time-step entropy—to effectively quantify the uncertainty and complexity of network topology at different time points. Simultaneously, by integrating DTWRE features with high-dimensional node embeddings generated by Node2Vec and utilizing GraphSAGE to construct a spatiotemporal fusion modeling framework, the model achieves precise prediction of link formation and key node identification in public opinion dissemination. The model was validated on multiple public opinion datasets, and the results indicate that, compared to baseline methods, it exhibits significant advantages in several evaluation metrics such as AUC, thereby fully demonstrating the effectiveness of the dynamic time-weighted mechanism in capturing the temporal evolution of public opinion dissemination and the dynamic changes in network structure.

## 1. Introduction

The scale-free nature of social networks and the cascading effect of information dissemination have made them a central medium for the evolution of public opinion in modern society. With the exponential growth in network scale, the complexity of information dissemination paths has increased significantly. Research indicates that more than 78% of information spread on social networks adheres to a power-law distribution [1], and this uneven mode of propagation allows public opinion on sudden incidents to cascade network-wide within hours; notably, during emergent public health events, the spread rate of misinformation can be as much as six times higher than that of accurate information [2]. Given that the creation and spread of misinformation occur with astonishing rapidity, they can rapidly affect the sentiments and actions of many users, which may not only trigger social panic and mislead public decisions but also damage the reputations of governments or businesses, ultimately resulting in economic and social instability [3,4].

In contrast to conventional media channels, the decentralized characteristics of social networks enable every user to become a source of information, creating an environment conducive to the proliferation of misinformation. The underlying mechanism of viral rumor diffusion encompasses not only the heterogeneity in user behavior [5] but is also intricately linked to the dynamic changes in network structure entropy [6,7] and the evolution of community organization [8]. In public opinion propagation modeling, existing approaches have evolved through three stages: early studies primarily employed differential equation models based on propagation dynamics [9], but their oversimplified assumptions regarding network structure limited predictive accuracy; intermediate graph network approaches enhanced modeling capabilities by incorporating community structure and node centrality [10], yet they failed to address the integration of dynamic features; and recent machine learning techniques have achieved breakthroughs using graph embedding [11,12] and temporal modeling [13], although challenges remain in managing the causal relationships [14] and heterogeneity [15] of propagation paths.

In recent years, the application of graph entropy has extended beyond merely measuring network complexity to include predicting information propagation paths and identifying key nodes [16]. In particular, in scenarios such as public opinion dissemination [17] and virus transmission [18], graph entropy helps in understanding and forecasting the interactions among nodes and their information dissemination processes. Serving as a metric for network complexity, graph entropy is capable of depicting the global and local attributes of networks through the lens of information theory. Dehmer and Emmert-Streib investigated the application of graph entropy in complex networks, suggesting that by measuring characteristics like network topology and node distribution, graph entropy effectively captures network complexity [19]. While static network analyses utilizing Shannon entropy [20] can characterize structural stability, they are limited in their ability to portray dynamic evolutionary processes. As a generalized form of information entropy, Rényi entropy demonstrates distinct advantages in accommodating diverse network topologies; however, its application in public opinion propagation forecasting remains underexplored.

Additionally, numerous investigations have started leveraging machine learning—especially graph-based deep learning techniques—to model and forecast information spread in networks, thereby overcoming the limitations of conventional public opinion dissemination models in coping with complexity and dynamism. Graph neural networks (GNNs) have emerged as a significant breakthrough in network-structured data analysis in recent years. Notably, GraphSAGE (Graph Sample and Aggregation) stands out for its excellent inductive learning ability by aggregating features from neighboring nodes to learn node representations, thereby enabling the handling of large-scale graph data [21]. Ref. [22] introduced a GraphSAGE-based dynamic spatiotemporal graph convolutional network for traffic forecasting, which adeptly captures the dynamic spatiotemporal interdependencies in traffic networks and improves prediction precision. When applied to public opinion dissemination prediction, GraphSAGE effectively detects inter-node relationships and propagation potentials, especially in social networks where it manages network heterogeneity, node attributes, and structural information. The authors of [23] employed a weighted version of GraphSAGE to develop a rumor detection model called GSMA, which significantly improved the accuracy of rumor detection on the Weibo platform. In [24], the researchers introduced a temporal graph neural network (TGN) model that effectively analyzes the dynamic variations in the process of information propagation.

Moreover, by combining features, including graph structure, transmission chains, and user behavior metrics [25], researchers have further enhanced the predictive performance of their models. The authors of [26,27] profoundly considered the limitations of traditional methods in measuring the importance of nodes in complex networks. Based on local and global information, respectively, they constructed the LSS algorithm and the GSM model, achieving significant progress in the field of complex networks. However, although the above-mentioned methods have made significant progress in the utilization of graph structure and node relationships, how to extract and fuse the features of the graph more comprehensively and effectively remains the key to improving the performance of the model. In order to improve both the predictive capability and stability of the model, this research incorporates graph embedding methods to achieve feature fusion. The Node2Vec algorithm [28], as a graph embedding method, generates neighborhood information for nodes through random walks, thereby learning low-dimensional representations that offer a novel perspective for analyzing complex networks. Within graph neural networks, the node embeddings produced by Node2Vec are used as input features, transforming intricate network structures into a format accessible to machine learning models, thereby aiding in the effective capture of latent relationships between nodes [29]. This approach has seen extensive application in public opinion propagation and link prediction tasks.

Current research on public opinion dissemination prediction faces key challenges. Firstly, existing models are unable to adequately consider the temporal dynamic characteristics of network-based public opinion propagation—traditional models, largely based on static network assumptions [20,30], struggle to capture the time-varying nature of node interactions. Although graph embedding techniques optimize node representations through random walk strategies, they remain insufficient in modeling time decay effects [31] and causal relationships [32]. Secondly, assessments of node influence depend excessively on static indicators like degree centrality [33] and tend to concentrate on local node characteristics, overlooking the quantifiable contribution of dynamic entropy in forecasting propagation routes. For instance, the entropy-based influence model proposed in [34] can capture interaction frequency characteristics, yet it fails to consider the topological evolution along the temporal dimension. Thirdly, the limitations of feature integration are significant: Most existing methods focus solely on unidimensional features, such as textual sentiment analysis [3] or community structural characteristics, while failing to effectively integrate multidimensional information encompassing user behavioral traits, social relational attributes, and temporal propagation dynamics. Consequently, these models struggle to accurately forecast the propagation trajectories and scope boundaries of public opinion dynamics amidst entangled multi-factorial influences in real-world scenarios.

To address the aforementioned challenges, our research introduces a network public opinion dissemination prediction model that leverages dynamic time-weighted Rényi entropy (DTWRE) alongside deep learning. By fusing node time-weighted Rényi entropy features with Node2Vec embeddings, the model forms an innovative spatiotemporal fusion framework based on graph entropy theory and deep learning. Its core advantage lies in combining network topology with propagation complexity (that is, the inherent spatio-temporal heterogeneity and uncertainty of public opinion dissemination), which enables a more comprehensive characterization of public opinion dissemination patterns. This integration not only enhances the accuracy of public opinion dissemination prediction but also accounts for temporal dynamics, rendering the prediction results more interpretable and practically valuable. The graph neural network-based public opinion dissemination prediction model, by combining network structural information, node features, and propagation history data, can effectively uncover the underlying patterns in public opinion spread. Firstly, a dynamic Rényi entropy measurement system is constructed based on generalized graph entropy theory [35], introducing a time-weighting factor to jointly model network structural complexity and propagation dynamics. By tuning the entropy order α, this metric can flexibly capture the entropy variations present within diverse network structures and across various temporal windows. Secondly, by integrating the strengths of Rényi entropy features with those of graph embedding techniques during node embedding and by fusing network structure, node attributes, and historical dissemination data, a predictive model is developed that automatically uncovers key insights embedded in the network and effectively captures the dynamic evolution of complex network structures. Lastly, an assessment framework is constructed by integrating metrics, including node Rényi entropy, global Rényi entropy across time steps, and timeliness evaluations, which validates the model’s superior performance in detecting key nodes and predicting propagation paths.

In summary, the principal contributions of this research are as follows:

- The introduction of dynamic time-weighted Rényi entropy furnishes a quantitative metric to capture the temporal dynamics and intrinsic structural complexity of network dissemination.
- A dual-level Rényi entropy metric is devised at both local node and global time-step scales, thereby facilitating the integrated modeling of localized and global structural complexity alongside propagation dynamics.
- The confluence of propagation temporal complexity and graph topology is exploited to establish a comprehensive spatiotemporal fusion model.
- The model’s generalizability is rigorously validated across multiple real-world public opinion datasets, advancing innovative methodologies for key node detection and propagation pathway prediction.

## 2. Methods

This study presents a network public opinion propagation prediction model that integrates DTWRE with GraphSAGE. Its core concept is to quantify the varying complexity of the network structure at different time points using time-weighted Rényi entropy and to combine this entropy feature with node feature vectors derived from graph embedding techniques, which are subsequently input into a downstream graph neural network for link prediction tasks, ultimately achieving public opinion propagation forecasting. This section provides a detailed overview of the innovative methodologies employed in this research, including the computation of time-weighted Rényi entropy and the construction of an integrated GraphSAGE model framework.

### 2.1. Spatiotemporal Fusion Modeling

Within the domain of public opinion propagation prediction, Node2Vec employs random walks to learn the network’s topological information, effectively capturing both local and global node associations, as well as the structural characteristics of dissemination paths. Unlike DeepWalk, whose fixed random walk strategy limits its capacity to model heterogeneous propagation paths, Node2Vec’s biased search (via parameters p and q) enables flexible interpolation between breadth-first (BFS) and depth-first (DFS) sampling. This is critical for capturing both of the following:

- Local neighborhood structures (BFS-like), which reflect immediate opinion diffusion patterns.
- Macro-scale dissemination pathways (DFS-like), which model long-range cascading effects in social networks [12].

Although the low-dimensional vectors generated by Node2Vec retain substantial structural details, they inherently lack the capacity to represent the temporal evolution of public opinion, rendering them unable to distinguish between recently established and older connections. Moreover, a notable limitation of the Node2Vec algorithm is that its data-driven vector representations are difficult to interpret in terms of physical significance—such as node importance or dissemination capacity—a shortcoming that is effectively mitigated by incorporating Rényi entropy features.

The novel time-weighted Rényi entropy introduced herein quantifies the uncertainty inherent in node propagation by incorporating the temporal dimension, thereby deepening the model’s predictive capability and elucidating the evolutionary trends of public opinion dissemination. As a metric for assessing propagation uncertainty, Rényi entropy—when augmented by a tailored time-weighting mechanism—provides a refined portrayal of the dynamic complexity of nodes during public opinion dissemination. This approach further facilitates the modeling of the relative importance of different temporal phases, enabling the differentiation between early influencers and later disseminators and thereby advancing the spatiotemporal exploration of public opinion propagation.

**Figure 1:**
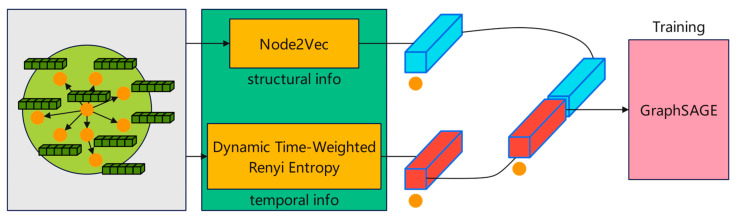
Schematic diagram of the spatiotemporal fusion modeling process.

However, a solitary entropy measure is insufficient to encapsulate the full spectrum of network structural characteristics, potentially yielding a lower informational content compared to the high-dimensional embeddings generated by Node2Vec. Consequently, by integrating the complementary strengths of both approaches, Node2Vec is employed to capture the global network topology and discern the topological nuances of dissemination pathways. The incorporation of time-weighted Rényi entropy introduces temporal dynamic information, enabling the model to effectively perceive the evolving patterns of public opinion dissemination over time. The neighbor sampling strategy of GraphSAGE reduces the computational complexity while maintaining the structural fidelity and is capable of simultaneously learning spatial topological relationships and temporal evolution trends—better aligned with the propagation mode of real social networks. The complete spatiotemporal fusion modeling process is illustrated in Figure 1. By leveraging the high-dimensional topological information from Node2Vec together with the temporal dynamic features of Rényi entropy, and training through GraphSAGE, the network structure and propagation characteristics are seamlessly integrated. This fusion enhances prediction accuracy, generalizability, and interpretability while improving model stability, ultimately yielding a superior predictive framework for social network public opinion analysis.

### 2.2. Definition of Dynamic Time-Weighted Rényi Entropy

Rényi entropy is a generalized entropy measure used to assess the uncertainty inherent in network structures. In this study, the Local Node Entropy (LNE) is computed using the Rényi entropy, with its mathematical formulation [36] defined as:

(1) $$H_{\alpha }(v,t)=\frac{1}{1-\alpha }\log\sum_{u\in N(v,t)}p_{u}^{\alpha }(t)$$

Here, N(v,t) denotes the set of neighbors for node v at time step t; $p_{u}^{\alpha }(t)$ represents the normalized probability distribution of an information metric (such as node degree or dissemination influence) for neighbor node u—with normalized probabilities derived from both node degree and PageRank values employed for comparative experiments—and α is the order parameter of Rényi entropy, which fundamentally governs its sensitivity to varying degrees of sparsity in probability distributions.

Mathematically, as α→0, the entropy emphasizes low-probability events (sparse connections), effectively capturing structural diversity in networks with heterogeneous propagation paths. Conversely, when α→∞, the entropy concentrates on high-probability events (dense hubs), which is pivotal for identifying influential nodes in scale-free networks. In our proposed DTWRE, α serves as a tunable parameter to balance these extremes. For instance, in scenarios like emergent public opinion dissemination, e.g., hot events on Weibo, information propagates through both central hubs and peripheral nodes. Setting α=0.6 (as validated in Section 4.2.1) optimally weights sparse and dense interactions, thereby enhancing the detection of latent propagation pathways. This adaptability enables DTWRE to dynamically align with the heterogeneous characteristics of real-world social networks.

To enhance predictive performance, a temporal factor is introduced via a time-weighting function ω(t) that aggregates entropy values from different time snapshots, thereby ensuring that more recent dissemination information exerts a greater influence on the overall entropy—closely mirroring the mechanisms of actual public opinion propagation. The proposed Dynamic Time-Weighted Rényi Entropy (DTWRE) is computed as:

(2) $$H_{\alpha }^{global}(t)=\sum_{v\in V_{t}}H_{\alpha }(v,t)$$

(3) $$H_{\alpha }^{time}(G,t)=\sum_{t_{k}<t}\omega (t-t_{k})H_{\alpha }^{global}(t_{k})$$

Here, $H_{\alpha }^{global}(t_{k})$ indicates the entropy of the network snapshot at time $t_{k}$. $\omega (t-t_{k})$ is the time weight function, using exponential attenuation weight:

(4) $$\omega (t-t_{k})=e^{-\lambda (t-t_{k})}$$

Here, λ > 0 governs the contribution of each time step to the final entropy value; a larger λ emphasizes recent dissemination, whereas a smaller λ allows for consideration of public opinion propagation over a more extended temporal range.

Based on the definition of Rényi entropy, two entropy indicators—local node entropy and global time-step entropy—have been designed to characterize the multiscale information complexity inherent in public opinion propagation. These indicators are subsequently employed as features for training the GraphSAGE model. The algorithm of the overall research method is shown in Algorithm 1. DTWRE comprehensively accounts for both the propagation characteristics of individual nodes and the influence of the network’s cumulative dissemination history on its current state, thereby providing an effective metric for modeling the complexity of public opinion propagation. Subsequently, LNE and DTWRE will be integrated with Node2Vec embedding features to furnish the downstream learning model with enriched feature inputs.
**Algorithm 1:** DTWRE metric based on time weighting and time step segmentation.
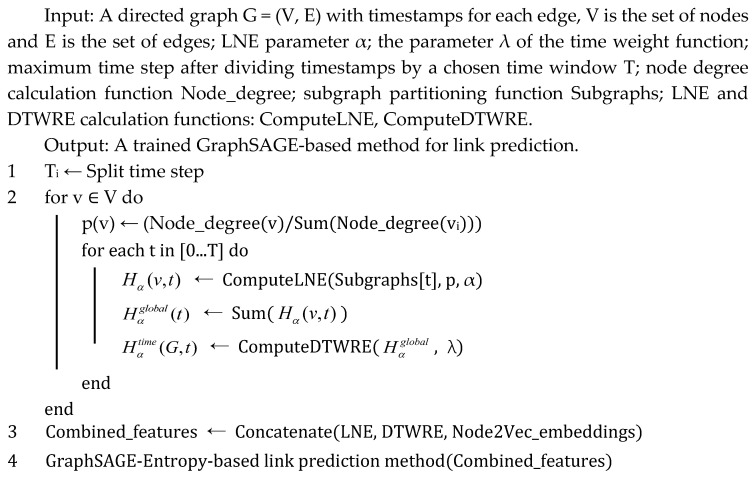


### 2.3. GraphSAGE for Public Sentiment Prediction

To leverage graph-structured information for public opinion propagation forecasting, the GraphSAGE framework is employed for this task. Among the prevalent graph neural network architectures, Graph Convolutional Networks (GCNs) and GraphSAGE are two pivotal models. Both models are designed to extract meaningful patterns and features from graph data by training on extensive datasets, thereby acquiring parameters that enable accurate predictions across various graphs, facilitating tasks such as node classification and link prediction through comprehensive processing of node and edge information.

GCNs learn vertex embeddings by integrating topological structure with vertex attribute information. However, GCNs require learning embeddings within a fixed graph and cannot directly generalize to vertices unseen during training—a hallmark of transductive learning. This necessitates retraining the model when new nodes emerge, leading to high computational costs and rendering such approaches less suitable for dynamic or large-scale graph data.

**Figure 2:**
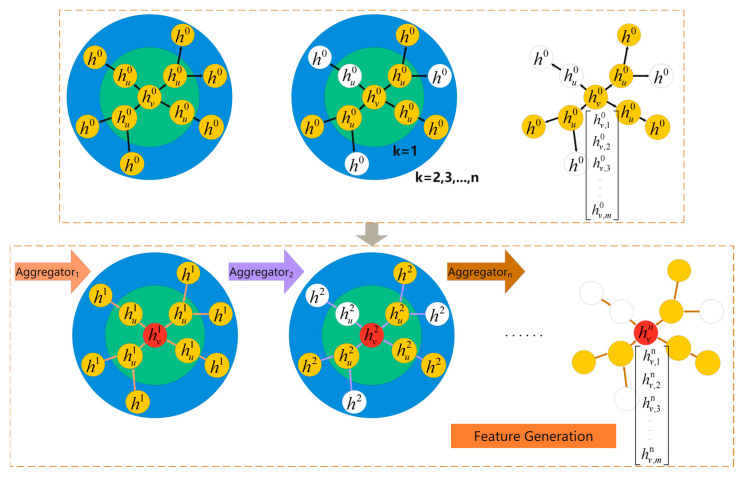
Schematic diagram of the GraphSAGE sampling and aggregation process.

In contrast, GraphSAGE constitutes an inductive learning framework capable of efficiently generating embeddings for unseen vertices by leveraging vertex attribute information. Its core idea is to learn a function that aggregates the representations of neighboring vertices to generate the embedding vector of the target vertex. This is of vital importance for dynamic networks where new users keep emerging. The specific workflow is shown in Figure 2. The principal features of this approach include:

1. Neighbor Sampling: Given that social networks typically manifest as large-scale sparse graphs, GraphSAGE randomly samples a fixed number of neighbors for each node to curtail computational complexity—an essential strategy for managing extensive graph datasets.
2. Feature Aggregation: For each node u, GraphSAGE aggregates features from its neighboring nodes and updates its own representation accordingly, as delineated in the following formulation:

(5) $$h_{u}^{\left(k\right)}=\sigma (W_{k}\cdot Aggregate(\{h_{v}^{\left(k-1\right)},\forall v\in N(u)\}))$$

where $h_{u}^{\left(k\right)}$ is the feature representation of node u at layer k. Aggregate{} is an aggregation function, where mean aggregation is used. $W_{k}$ is a trainable parameter. σ is a nonlinear activation function, where ReLU is used.

3. Final representation: After multi-layer aggregation, the final representation of node v is:

(6) $$h_{v}^{\left(n\right)}=[h_{v,1}^{n},h_{v,2}^{n},h_{v,3}^{n},\ldots ,h_{v,d}^{n}]$$

In this study, the prediction of public opinion propagation is modeled as a dynamic link prediction task. We incorporate DTWRE as an additional feature into the input of GraphSAGE, enabling the model to learn the temporal dynamic characteristics during the propagation process. As the core component for aggregating spatiotemporal features in the prediction process, GraphSAGE aims to jointly encode the dynamic topological characteristics and temporal evolution characteristics by learning node embeddings and ultimately achieves accurate prediction of public opinion propagation paths and key nodes. For the specific application of GraphSAGE in this study, please refer to Section 3.3.

## 3. Experiments

This section delineates the experimental procedures and results of the proposed deep learning-based public opinion propagation prediction model, which integrates DTWRE with Node2Vec embeddings, evaluated on real-world data. The experiments were conducted on the PyCharm platform, utilizing development tools such as PyTorch and the Deep Graph Library (DGL). DGL is a deep learning framework specifically designed for graph neural networks, offering efficient, flexible, and user-friendly tools for GNN research and development. To comprehensively assess the model’s performance, comparisons were made with existing link prediction methods, demonstrating its superior results across various evaluation metrics.

### 3.1. Datasets and Data Preprocessing

For evaluation purposes, the following datasets were employed during both the validation and empirical phases:

CollegeMSG Dataset: A classic dataset from SNAP comprising private messages exchanged on an online social network. As a temporal network widely used in research, it was employed during the validation phase to facilitate performance comparisons with other methodologies.

Real-world Social Network Dataset: Chinese rumor data, including repost and comment information, scraped from the Sina Weibo misinformation reporting platform. Predicting these rumors aids in characterizing the propagation patterns of misinformation, thereby providing theoretical support for combating false information on social networks.

**Figure 3:**
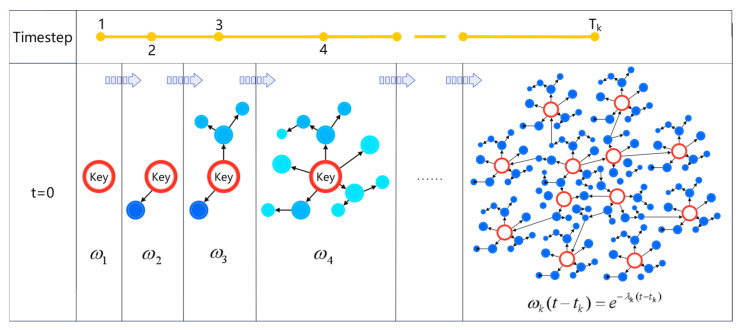
Schematic diagram of dynamic time weighting mechanism.

The following data preprocessing steps were applied to the aforementioned raw datasets:

▪ Time labels were standardized by converting them into timestamp format.
▪ Given the scale of real-world social network datasets, we implement data augmentation by introducing node-edge perturbations within each temporal window. Specifically, we randomly add 5% of nodes and edges and delete 2% of nodes and edges to simulate network uncertainty while simultaneously mitigating data sparsity issues.
▪ Isolated nodes, which do not participate in information dissemination, were removed to ensure graph connectivity and to uphold the validity of Rényi entropy computations.
▪ From the real-world social network dataset—which includes the content of rumor-related Weibo posts, the publishers, records of reposts and comments, and interaction timestamps—filtering was conducted to extract the rumor originators along with the corresponding interaction relationships (comments and reposts) to construct a public opinion propagation network.
▪ Temporal window segmentation was performed by partitioning dissemination data into multiple time windows based on timestamps; each window corresponds to a time step with gradually varying weights. Different datasets were segmented into time steps according to empirical criteria. For the CollegeMSG dataset, spanning 193 days, we adopted a 7-day sliding window to balance temporal granularity and computational efficiency, as validated through experiments in Section 4.2.4. For real-world social datasets, where interactions occur 1–N times per minute, a 1 h window was selected to reconcile longitudinal coverage with operational feasibility. The division diagram is shown as Figure 3.

The objective of the public opinion propagation prediction task is to forecast potential future links based on the known network structure. The ratio of positive to negative samples impacts both model training and generalizability. The construction of these samples is as follows:

- Positive samples: Connected node pairs (u, v) extracted from the actual network.
- Negative samples: A negative sampling strategy was employed to randomly select unconnected node pairs (u, v) from the set of non-existent edges.

Here, E denotes the set of actual edges in the network.

However, real-world networks are typically sparse—with the majority of potential edges absent—posing challenges for negative sample selection. Inadequate negative sampling can lead to data imbalance and diminish the model’s discriminative power. To address this issue, oversampling was applied to the negative samples.

To ensure the temporal rationality of the model evaluation, we adopted a training set (80%), a validation set (10%), and a test set (10%) that were strictly partitioned in chronological order. That is, the alignment is carried out according to the time window: the original data are sorted in ascending order of the timestamp, and the experimental dataset is constructed using “80% of the total duration and 90% of the total duration” as the demarcation points.

### 3.2. Feature Engineering

In this study, a multidimensional feature set is constructed to comprehensively capture both the dynamic information and structural characteristics inherent in public opinion propagation, serving as the input to the GraphSAGE model. The primary features utilized are categorized into two groups: entropy-based features and node embedding features.

#### 3.2.1. Rényi Entropy Feature

As previously described, these features encompass both local and global dimensions. Local Node Entropy (LNE) quantifies a node’s propagation potential and local structural complexity within a given time step—each node v is characterized by its LNE at time t—while Dynamic Time-Weighted Rényi Entropy (DTWRE) measures the overall network complexity at a specific time step. Their integration yields a holistic evaluation of the complexity of public opinion dissemination across various propagation stages.

#### 3.2.2. Node2Vec Embedding Features

To further capture the semantic and structural relationships among nodes, the Node2Vec algorithm is employed to generate node embeddings, following these specific steps:

1. Graph Conversion: Transform the graph constructed with DGL into NetworkX format and convert it into an undirected graph to satisfy the requirements of Node2Vec.
2. Embedding Computation: Perform random walk sampling on the network using the Node2Vec model and generate continuous vector representations for nodes via the Skip-Gram model, with the embedding dimension set to 64.
3. Normalization: To eliminate scale discrepancies, normalize the generated node embeddings using MinMaxScaler and convert them into Tensor format to ensure consistency with other features.

These node embedding features effectively complement the limitations of entropy-based metrics in capturing local structural nuances and semantic relationships, thereby enriching the node representations for the link prediction task.

#### 3.2.3. Feature Fusion

In the final feature construction phase, the LNE, DTWRE, and Node2Vec embeddings are concatenated to form a comprehensive feature vector.

This fusion approach integrates multiscale information: LNE captures the uncertainty of individual nodes within local structures, DTWRE reflects the network’s overall complexity at each time step, and node embeddings supply high-dimensional semantic details among nodes. Moreover, the incorporation of a time-weighting strategy allows the model to flexibly account for the influence of historical dissemination on the current state, thereby enhancing the capture of temporal dynamics in public opinion propagation. The resultant composite feature vector provides the GraphSAGE model with a more comprehensive input, facilitating more precise discrimination between positive and negative samples in link prediction and ultimately improving predictive accuracy.

### 3.3. Model Construction and Training

The detailed process for model construction and training is outlined below:

1. Model Architecture: The proposed model is comprised of two principal components:
  (1) Feature Input Layer: This layer ingests the extracted node features—both entropy-based and embedding features—as input.
  (2) Graph Neural Network Layer: Employing GraphSAGE, this layer aggregates features from neighboring nodes to update node representations, thereby generating embeddings for nodes across distinct time windows.
2. Predictor and Loss Function: To facilitate effective link prediction, a multilayer perceptron (MLP) is employed as the predictor. Comprising multiple fully connected layers, the MLP is designed to capture nonlinear relationships among node features and ultimately outputs a prediction score that quantifies the likelihood of a link forming. The binary cross-entropy loss function is used to optimize the predicted link probabilities for node pairs. Specifically, given a node pair (u, v), the model forecasts whether these nodes will establish a connection within a future time window. The formulation is as follows:

(7) $$L=-\sum_{(u,v)\in E}y_{uv}\log({\hat{y}}_{uv})+(1-y_{uv})\log(1-{\hat{y}}_{uv})$$

where $y_{uv}$ denotes the true connectivity status of node pair (u, v) and ${\hat{y}}_{uv}$ represents the model’s predicted link probability.

3. Training Process: The training phase employs the Adam optimizer—recognized for its adaptive learning rate and rapid convergence—with hyperparameters refined via cross-validation. The model is trained over 100 epochs to minimize the loss function, thereby achieving the improvement of performance.

### 3.4. Evaluation Metrics

To facilitate comparisons with other approaches and to comprehensively assess the model’s performance, the following commonly used evaluation metrics are adopted:

AUC (Area Under the ROC Curve): The probability that a randomly chosen positive sample is ranked higher than a negative sample. This metric gauges the model’s ability to differentiate between positive and negative samples; higher values indicate superior predictive performance.

Precision: Precision reflects the proportion of correctly predicted positive node pairs among those predicted as positive, with higher precision denoting greater accuracy.

(8) $$Precision=\frac{True\;Positives}{True\;Positives+False\;Positives}$$

Recall: Recall measures the proportion of actual positive node pairs that are successfully identified by the model, with higher recall indicating enhanced coverage of positive instances.

(9) $$Recall=\frac{True\;Positives}{True\;Positives+False\;Negatives}$$

F1-Score: The F1-score, defined as the harmonic mean of precision and recall, provides a balanced evaluation of the model’s predictive capability.

(10) F1-Score=2×Precision×RecallPrecision+Recall

Accuracy: Predicts the proportion of correct samples in the total sample.

(11) $$Accuracy=\frac{True\;Positives+True\;Negatives}{True\;Positives+True\;Negatives+False\;Positives+False\;Negatives}$$

Temporal Performance: Considering the pronounced temporal evolution in public opinion propagation, the model’s performance is assessed across varying time steps to monitor accuracy fluctuations over time.

**Figure 4:**
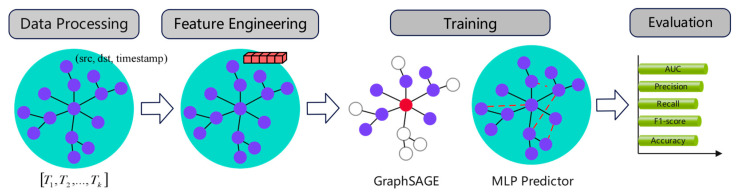
Experimental procedure of the innovative model on real-world public opinion datasets.

In summary, the key experimental steps encompass data processing, feature engineering, model training, and evaluation, as illustrated in Figure 4.

### 3.5. Experimental Parameter Setting

During both training and evaluation, extensive experimental configurations and hyperparameter tuning were conducted to optimize model performance. The following parameters were systematically refined:

1. Temporal Window Size: This parameter affects the model’s ability to learn sequential information. Empirically determined window sizes (as detailed in Section 3.1) were applied for different datasets to more effectively capture the dynamic nature of public opinion propagation.
2. α Value (Entropy Order for LNE): This parameter determines the flexibility of the entropy calculation. A grid search approach was utilized to optimize α and select its optimal value.
3. λ Value (Temporal Weighting Parameter): λ was tuned based on the characteristics of different time steps and historical public opinion propagation data; that is, the candidate set including those covering short-term advantages (λ > 1) and long-term historical influences (λ < 1) was selected to search for the optimal value.
4. Negative Sampling Ratio: A specified proportion of negative samples was drawn during training. Various ratios were experimented with to evaluate their impact on model performance.

To validate the effectiveness of the proposed approach, multiple baseline models were implemented for comparative analysis against the following classical prediction methods:

① Traditional Node Degree-Based Method;
② Traditional Node PageRank-Based Method;
③ Node2Vec-Based Method Utilizing Node Embeddings;
④ Traditional Static Rényi Entropy-Based Method.

These baselines effectively corroborate the innovation of the proposed method while providing a balanced comparison of the contributions of various features to overall model performance.

## 4. Results

This section delineates the experimental findings of the proposed network public opinion propagation prediction model, which integrates DTWRE with deep learning, as evaluated on benchmark datasets. Through comparative experiments, performance assessments, temporal analyses, extensions using real-world public opinion data, and time complexity analysis, the efficacy of the proposed approach in predicting network public opinion propagation has been validated, with a detailed analysis of the various factors influencing model performance. The results indicate that the graph neural network model augmented with DTWRE attains higher accuracy and superior temporal responsiveness compared to traditional approaches.

### 4.1. Model Performance Comparison

The experiment compared the four baseline methods mentioned above, and the comparison results of evaluation indicators are as follows.

**Figure 5:**
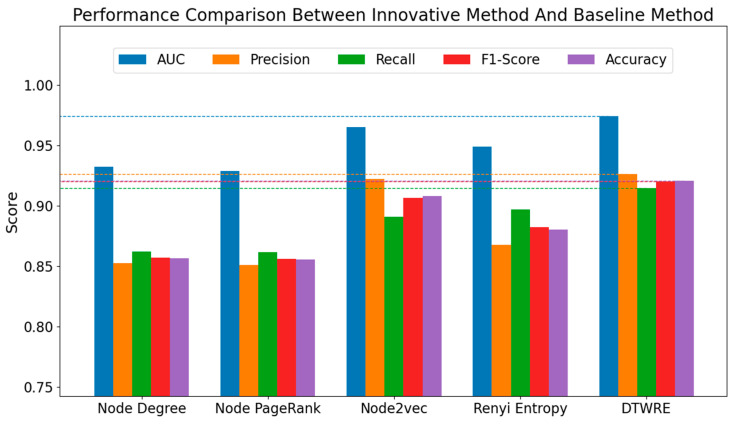
Performance comparison between innovative method and baseline method.

According to the findings in Table 1 and Figure 5, the innovative DTWRE method achieved the highest AUC (0.9742), demonstrating its capacity to better capture the latent dynamic propagation characteristics within the network, thereby enhancing its discriminative ability in distinguishing positive from negative samples. Moreover, DTWRE attained the highest precision at 0.9259, indicating that the vast majority of predicted positive samples correspond to genuine links. This outcome further confirms the effectiveness of node embeddings in capturing semantic and structural nuances while also highlighting the limitations of relying solely on embeddings to fully represent the dynamic aspects of public opinion propagation. In terms of recall, both DTWRE (0.9144) and Rényi entropy (0.8970) outperform methods based on node degree (0.8618) and PageRank (0.8613), indicating that the incorporation of entropy features enables a more comprehensive detection of existing links, thereby reducing false negatives. The novel approach achieved the highest F1-score (0.9201), signifying an optimal balance between precision and recall. When compared to Node2Vec’s F1-score (0.9062), this result corroborates the superior overall performance conferred by the time-weighted mechanism. With an accuracy of 0.9207, DTWRE consistently outperforms all alternative methods, thereby demonstrating its robust performance in comprehensive link prediction tasks.

In summary, given that the influence of information at various time steps on network structure is heterogeneous during public opinion propagation, Rényi entropy enriches the dissemination information available to nodes, thereby enabling a more effective capture of latent structural features. Furthermore, time-weighted entropy features adeptly adjust to the evolving characteristics of public opinion propagation, enhancing the model’s performance in dynamic dissemination scenarios. The proposed DTWRE method, by incorporating exponential decay weights to effectively integrate information across different time steps, surpasses all baseline methods across various metrics, which shows its efficacy and state-of-the-art performance in network public opinion propagation prediction.

### 4.2. Model Parameter Analysis

To further elucidate the impact of key parameters on link prediction performance, extensive experiments were conducted on the DTWRE order α, the temporal weighting factor λ, and the positive-to-negative sample ratio, with the effects of varying these parameters on model performance illustrated via comprehensive visual charts.

#### 4.2.1. Impact of DTWRE Order α

We explore the effect of DTWRE order α on the model performance by changing it. The consequences are as follows.

**Figure 6:**
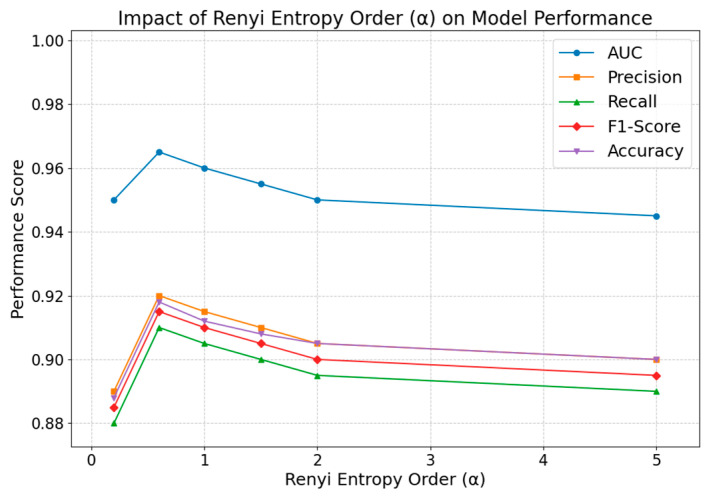
Impact of DTWRE order α on model performance.

Figure 6 illustrates that when α is set to 0.2, the AUC reaches 0.950, indicating a relatively low sensitivity in the entropy calculation. This low sensitivity overemphasizes sparse connections, resulting in an inability to fully capture the structural diversity among neighboring nodes. As α increases to 0.6, the AUC sharply ascends to 0.966, demonstrating that the entropy metric more effectively reflects the distributional uncertainty among node neighbors, thereby enhancing the model’s ability to differentiate between positive and negative samples. With further increases in α to 1, 1.5, 2, and 5, the AUC declines to 0.959, 0.955, 0.952, and 0.944, respectively, suggesting that excessively high α values overly accentuate the high-probability components of the distribution while neglecting lower-probability information, which in turn degrades the overall discriminative capability. Precision peaks at 0.922 when α is 0.6, followed by a slight decline as α increases further. The trends in recall and F1-score mirror that of precision, achieving their optimum at α = 0.6, while both lower and higher values of α fail to attain an optimal balance. This variation indicates that an α value of 0.6 allows the entropy measure to effectively capture the diversity of a node’s propagation potential, thereby enabling more precise discrimination between positive and negative samples; conversely, values that are too low or too high tend to induce information loss or introduce noise, thereby impairing overall performance. The accuracy trend parallels that of the AUC, rising from 0.888 at α = 0.2 to 0.916 at α = 0.6, and then gradually falling to 0.904 at α = 5, further substantiating that an optimal α value is 0.6.

Consequently, it can be concluded from the results that all performance metrics peak when α is 0.6, which aligns with the anticipated advantages of DTWRE features in capturing the dynamics of public opinion propagation. These data variations highlight the distinctive role of entropy in elucidating node influence and propagation uncertainty, offering a more comprehensive depiction of the complexity inherent in public opinion diffusion compared to conventional metrics that solely consider node degree.

#### 4.2.2. Impact of the Temporal Weighting Parameter λ

The parameter λ within the time-weighting function—an integral innovation of the DTWRE approach—warrants examination regarding its effect on model performance. The results are as follows.

**Figure 7:**
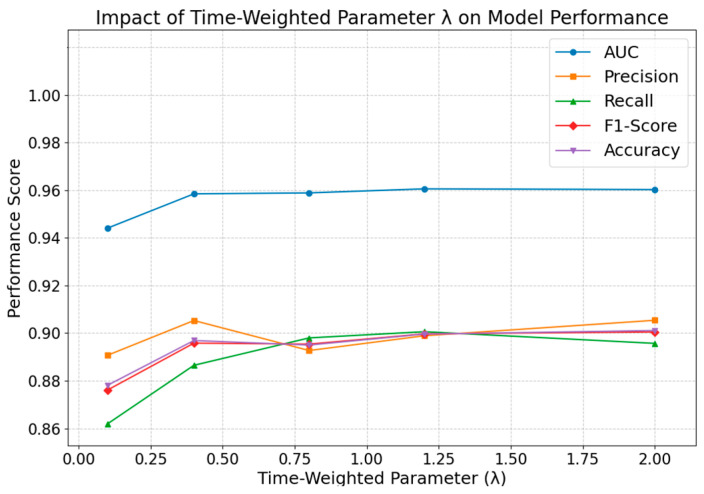
Impact of time-weighted parameter λ on model performance.

Figure 7 presents a comparative analysis of experimental data obtained under varying values of λ, revealing the following trends: When λ is set to a low value (e.g., 0.1), the insufficient emphasis on historical information results in overall suboptimal model performance; as λ increases from 0.1 to moderate values (ranging from 0.4 to 1.2), all performance metrics exhibit a marked improvement, indicating that moderately amplifying the weight of recent information facilitates the capture of dynamic public opinion propagation characteristics; at λ = 1.2, the model attains optimal levels in AUC, recall, F1-score, and accuracy, thereby achieving the best overall performance; when λ is further increased to 2, although precision and accuracy experience a modest rise, recall declines slightly, and overall metrics remain relatively stable, suggesting that an excessively high λ may lead to an overemphasis on recent data at the expense of historical information.

#### 4.2.3. Impact of the Positive-to-Negative Sample Ratio

**Figure 8:**
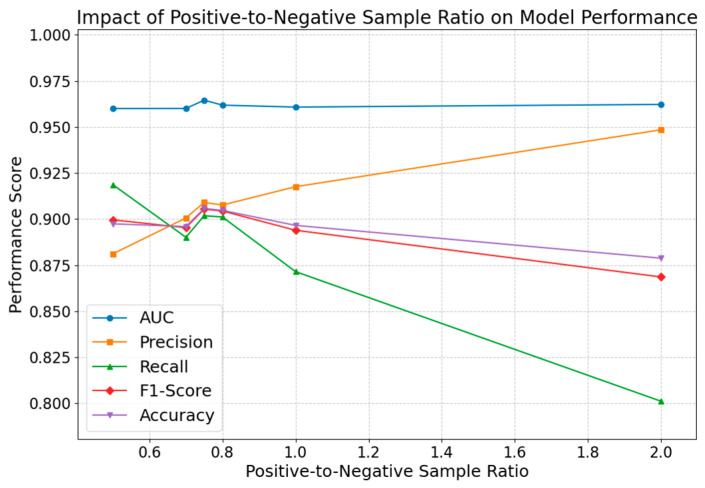
Impact of positive-to-negative sample ratio on model performance.

In this study, careful consideration is given to the positive-to-negative sample ratio, since severe imbalance may bias the model toward the predominant class, whereas an optimal ratio facilitates the effective learning of intrinsic data patterns and enhances overall model performance and predictive accuracy. The figure below (Figure 8) illustrates the impact of varying positive-to-negative sample ratios on performance metrics.

Analysis of Figure 8 reveals that the positive-to-negative sample ratio exerts a significant influence on model performance. When the ratio is set to 2—indicating an excess of negative samples—precision is high, yet recall declines markedly, resulting in lower overall F1-score and accuracy; conversely, when the ratio is set to 0.5 (fewer negative samples), recall is elevated but precision is insufficient. The optimal positive-to-negative sample ratio is approximately 0.75, at which point the model demonstrates the best performance in AUC, F1-score, and accuracy, thereby achieving an ideal balance between positive and negative samples.

#### 4.2.4. Temporal Performance Evaluation

To validate the model’s ability to capture the temporal dynamics of public opinion propagation, experiments were designed with varying time step lengths. Specifically, under constant conditions for other parameters such as λ, α, and the positive-to-negative sample ratio, the effect of different time step lengths on model performance was compared. The experimental results presented in Table 2 display performance metrics corresponding to time steps of 1.75 days, half a week, and one week.

**Figure 9:**
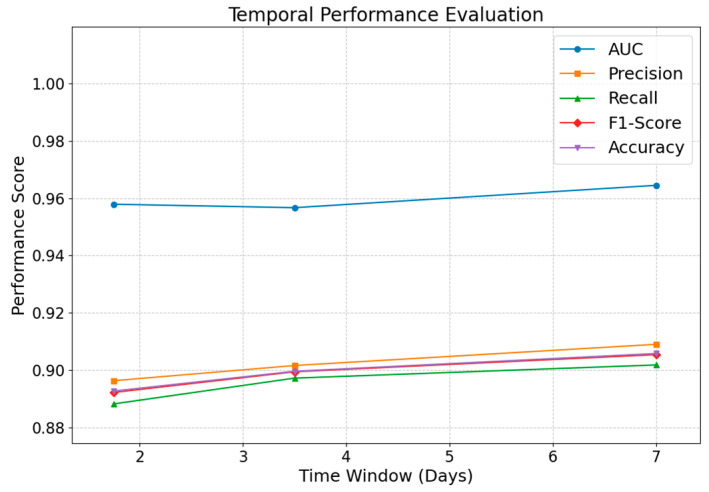
Temporal performance evaluation results.

As depicted in Figure 9, when the time window aligns with the overall temporal span of the dataset, employing a longer window offers a distinct advantage. Specifically, when a time window of 604,800 s (7 days) is employed, all performance metrics significantly outperform those obtained with shorter windows of 302,400 and 151,200 s. This indicates that a 7-day window can effectively capture the dynamic evolution of public opinion propagation and supply ample information for predicting link formation, whereas shorter windows may yield insufficient data, thereby diminishing overall performance. It is noteworthy that the 7-day window was determined based on the dataset’s total temporal span and empirical considerations; thus, only three appropriate window lengths were examined. Consequently, from a temporal performance standpoint, employing a longer time window is more conducive to robustly capturing the dynamics of network propagation.

### 4.3. Prediction of Real Social-Network Public Opinion

Following the model’s excellent performance on the benchmark dataset CollegeMSG, further analysis was conducted on a real-world Weibo dataset to assess its generalizability in capturing public opinion propagation. This dataset encompasses interactive information such as reposts and comments related to rumors among Weibo users. To investigate the dissemination mechanisms of false public opinion, the innovative advantages of the DTWRE method were leveraged to analyze the rumor data, with the results presented as follows.

**Figure 10:**
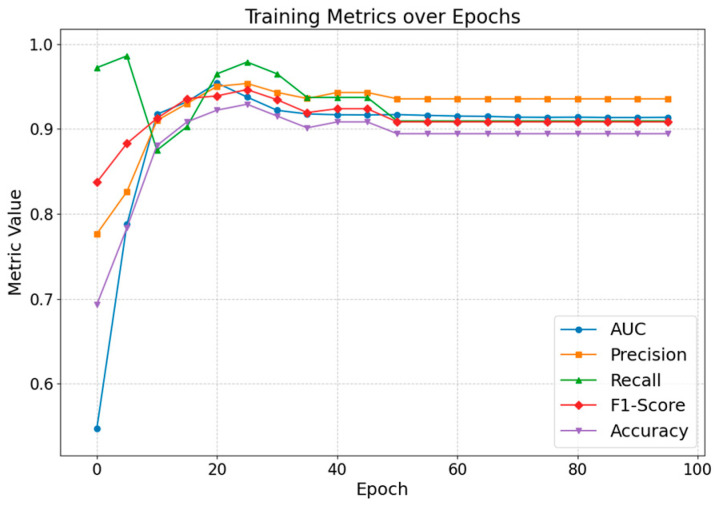
Results of the variation of performance metrics of the model in 100 training epochs.

Figure 10 illustrates the evolution of performance metrics over training epochs for the real-world public opinion dataset. Overall, the performance curves exhibit a rapid initial ascent, then gradually converge and stabilize around the 50th epoch at relatively high levels, reflecting the model’s robust capability to comprehensively distinguish between positive and negative links (i.e., public opinion propagation relationships) in the dataset.

**Figure 11:**
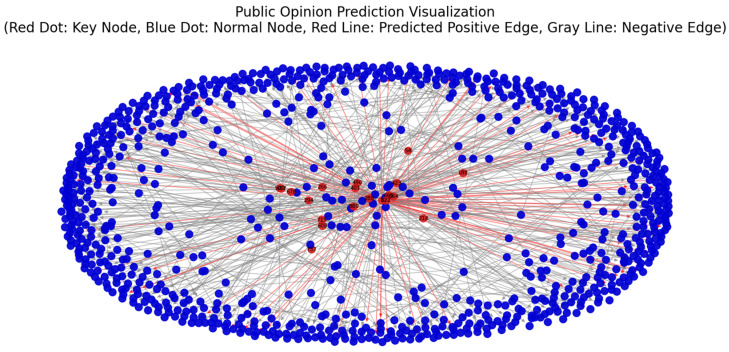
Visualization of model prediction results in real social network public opinion datasets.

Figure 11 reveals a “center-periphery” structure within the social public opinion network, where red nodes in the central region exhibit high entropy values and are identified as key nodes that form a densely connected core, rapidly disseminating information to peripheral nodes and warranting focused attention in public opinion management. The significance of these nodes is further validated by retrospectively tracing their diffusion pathways within the original dataset. Red lines denote links predicted as positive—indicating edges where actual connections or information flows are more likely to occur during public opinion propagation. It is evident that the red lines radiate outward from the center, confirming the model’s identification of diffusion pathways from key nodes to ordinary nodes.

### 4.4. Time Complexity Analysis

The propagation prediction framework integrating DTWRE and GraphSAGE proposed in this paper can be decomposed into the following core modules in terms of time complexity:

1. DTWRE Calculation:
  ● LNE: For each node υ and time step t, calculating $H_{\alpha }(\nu ,t)$ requires O(k) operations (where k is the average neighbor count). Total complexity: O(NTk).
  ● DTWRE: Aggregating $H_{\alpha }^{global}(t_{k})$ across T time steps with exponential decay introduces O(NT) complexity.
2. Node2Vec Embeddings: Random walks and Skip-Gram training have a complexity of O(rNl), where r is the number of walks per node, l is walk length.
3. GraphSAGE Training:
  ● Neighbor sampling and feature aggregation for each node involve $O(k^{d})$ operations per layer (depth d), leading to $O(Nk^{d})$ complexity.
  ● MLP predictor adds $O(D^{2})$ complexity (for D-dimensional features).

The overall time complexity of the framework is dominated by $O(NTk+rNl+Nk^{d})$, which remains feasible for large-scale networks due to parallelizable components (e.g., Node2Vec walks and GraphSAGE aggregation).

The comparison results of the time complexity of this research method with other research methods are shown in Table 3.

It can be seen from the above detailed analysis of the algorithm complexity and the results of the comparative experiments that the method proposed in this paper exhibits certain disadvantages in terms of time complexity. This is mainly attributed to the additional computational overhead introduced during the dynamic time modeling process, including operations such as the calculation of time windows based on DTWRE, the update of node time series features, and the calculation of time-sensitive node embeddings by combining Node2Vec and GraphSAGE. However, this additional overhead has brought about significant performance improvements. The experimental results on different real social network datasets show that this method can more accurately capture the temporal dynamic characteristics in the process of public opinion propagation. It has achieved more accurate and interpretable predictions of network public opinion propagation, providing a more reliable basis for the formulation of public opinion monitoring and intervention strategies.

## 5. Conclusions

This study addresses the critical issue of network public opinion propagation prediction by integrating machine learning techniques, complex network theory, and graph entropy analysis. A novel prediction model is proposed, which leverages dynamic time-weighted Rényi entropy to capture the evolving complexity of network dissemination and harnesses the benefits of Node2Vec graph embeddings. These features are jointly fed into a GraphSAGE-based graph neural network to facilitate accurate public opinion forecasting. Extensive experimental results demonstrate that the proposed method consistently outperforms existing approaches across multiple datasets. Its unique advantage is particularly evident when handling network data with temporal dependencies, leading to a significant enhancement in the accuracy of public opinion propagation predictions. Moreover, this research offers a novel perspective on public opinion propagation prediction and introduces an innovative approach for applying time-weighted entropy features in complex network analysis, thereby holding substantial theoretical and practical significance. Future investigations may extend the applicability of dynamic time-weighted Rényi entropy to various network environments—such as biological networks and recommendation systems—and explore the integration of additional node features and propagation mechanisms to further enhance model generalizability. In addition, incorporating multimodal data (e.g., textual content and social interaction metrics) into public opinion propagation prediction could yield more precise predictive models and robust decision support systems in relevant fields.

## Figures and Tables

**Table 1:** Comparison of the experimental results between the innovative method and the baseline method.

Baseline Methods	AUC	Precision	Recall	F1-Score	Accuracy
①	0.9323	0.8522	0.8618	0.8570	0.8562
②	0.9285	0.8509	0.8613	0.8561	0.8552
③	0.9649	0.9221	0.8909	0.9062	0.9078
④	0.9487	0.8677	0.8970	0.8821	0.8802
DTWRE	0.9742	0.9259	0.9144	0.9201	0.9207

**Table 2:** Impact of the time step length on temporal evaluation.

Time Step Length	AUC	Precision	Recall	F1-Score	Accuracy
604,800	0.9680	0.9159	0.9044	0.9101	0.9107
302,400	0.9567	0.9016	0.8972	0.8994	0.8996
151,200	0.9579	0.8963	0.8882	0.8922	0.8927

**Table 3:** Comparison of time complexity of each method.

Method	Time Complexity	Key Operation
This research	$O(NTk+rNl+Nk^{d})$	Entropy calculation, random walks, GNN
GCN	O(mdD)	Full-graph convolution (m: edges)
Node2Vec	O(rNl)	Random walks, Skip-Gram
DeepWalk	O(rNl)	Random walks, hierarchical softmax

## Data Availability

All data sets used in this research can be found on https://snap.stanford.edu/data/index.html (accessed on 29 April 2025) and https://github.com/yeren66/ChineseRumorDataset (accessed on 29 April 2025).

## Abbreviations

The following abbreviations are used in this manuscript:

DTWRE	Dynamic Time-Weighted Rényi Entropy
LNE	Local Node Entropy
